# Resveratrol Inhibits Oxidative Stress and Prevents Mitochondrial Damage Induced by Zinc Oxide Nanoparticles in Zebrafish (*Danio rerio*)

**DOI:** 10.3390/ijms21113838

**Published:** 2020-05-28

**Authors:** Roberta Giordo, Gheyath K. Nasrallah, Ola Al-Jamal, Panagiotis Paliogiannis, Gianfranco Pintus

**Affiliations:** 1 Biomedical Research Center, Qatar University, Doha P.O. Box 2713, Qatar; robertagiordo2000@yahoo.it (R.G.); ola.l.aljammal@gmail.com (O.A.-J.); 2 Department of Biomedical Science, College of Health Sciences, Member of QU Health, Qatar University, Doha P.O. Box 2713, Qatar; 3 Department of Medical, Surgical and Experimental Surgery, University of Sassari, Viale San Pietro 43, 07100 Sassari, Italy; ppaliogiannis@uniss.it; 4 Department of Medical Laboratory Sciences, College of Health Sciences and Sharjah Institute for Medical Research, University of Sharjah, Sharjah P.O. Box 27272, UAE; 5 Department of Biomedical Sciences, University of Sassari, Viale San Pietro 43, 07100 Sassari, Italy

**Keywords:** resveratrol, nanoparticles, ROS, oxidative stress, antioxidants, mitochondria, zebrafish, zinc oxide

## Abstract

Despite their wide industrial use, Zinc oxide (ZnO) nanoparticles (NPs) exhibit a high toxic potential while concerns of their health-related risks are still present, urging additional in vivo clarification studies. Oxidative stress is recognized as the primary trigger of NP-associated toxicity, suggesting antioxidants as a promising counteractive approach. Here, we investigated the protective effect of the natural antioxidant resveratrol against ZnO NP-induced toxicity in vivo using the zebrafish model. Our findings demonstrate that resveratrol counteracts ZnO NP-induced zebrafish lethality preventing cardiac morphological and functional damage. NP-induced vascular structural abnormalities during embryonic fish development were significantly counteracted by resveratrol treatment. Mechanistically, we further showed that resveratrol inhibits ROS increase, prevents mitochondrial membrane potential dysfunction, and counteracts cell apoptosis/necrosis elicited by ZnO NP. Overall, our data provide further evidence demonstrating the primary role of oxidative stress in NP-induced damage, and highlight new insights concerning the protective mechanism of antioxidants against nanomaterial toxicity.

## 1. Introduction

Nanoparticles (NPs) are particles of matter with a dimension between 1 and 100 nanometers (nm), comparable in size to subcellular structures and cell organelles. NPs properties (mechanical, optical, magnetic, electrical, and catalytic) are functions of their size, as they can widely vary at a parity of composition [1]. Moreover, physical and chemical properties can also depend on the NPs synthesis procedure [2]. Zinc oxide (ZnO) NPs are among the most utilized in different industrial applications, including cosmetic, biological, chemical, and construction [3]. Despite the wide industrial use, the labeling of ZnO NP-containing products is often confusing, making it difficult to understand the real/exact NPs presence in consumer products, and thus, obscuring the potential risk associated with them. Due to their notable ability to reflect UV and transparency to visible light, ZnO NPs are widely employed in sunscreen production [4]. Although human skin is an effective barrier to ZnO NPs, under particular conditions (e.g., abrasions), the very tiny dimension of NPs allows them to penetrate the skin and enter the body [4]. Airway exposure to NPs via inhalation is another potential risk for workers in the chemical, cosmetic, or paint industries. NPs may indeed reach and hurt the respiratory tract causing toxic effects and inflammatory responses in addition to an enhanced risk of developing cardiovascular diseases and cancer [5,6,7]. Studies employed in vitro show both cytotoxic and genotoxic damages [8,9]. Among the possible ZnO NPs toxicity associated mechanisms, increased production of reactive oxygen species (ROS) appears to play a central role [10]. Within the cell, oxidative stress results from the imbalance between ROS production and antioxidant defense [9]. When ZnO NPs enter the cell, the immune system and anti-inflammatory response produce increased ROS generation [10,11,12]. However, a prolonged or excessive ROS production is difficult to counterbalance by the antioxidant defense, leading, therefore, to oxidative stress [13,14]. In vivo animal studies also show that ZnO NPs exposure leads to antioxidant system impairment due to GSH, SOD, and CAT depletion [15,16,17]. An altered intracellular redox state also activates cytokines and chemokines release inducing pro-inflammatory responses leading to cell toxicity and tissue damage [14]. Further, redox imbalance-associated pathways can also cause DNA damage such as gene mutations, chromosomal fragmentation, and DNA strand breakages, ultimately inducing cell death by apoptosis or necrosis [14,17]. Since redox homeostasis plays a pivotal role in maintaining cellular physiology, the use of antioxidants might represent a potential approach to prevent or reduce NP-induced oxidative stress and its associated cell damage [14].

In this context, recent studies show that common antioxidants such as ascorbic acid and Vitamin E can reduce NPs toxicity associated with oxidative stress [18,19]. Natural occurring polyphenol compounds, including quercetin and curcumin, have also been reported to possess protective effects against NPs-induced oxidative damage [20,21,22]. Within phytochemicals, resveratrol, a compound occurring in many fruits like grapes, mulberries, peanuts, and cocoa, has been widely reported to exhibit antioxidant and scavenging activities [23]. In addition, emerging studies have reported a protective role of resveratrol against toxicity induced by nanomaterials [24] and ZnO NPs in epithelial cells, Nile tilapia, and Caenorhabditis elegans [25,26]. While there is a copious number of in vitro studies on the protective effects of antioxidants against NP-induced damage, there are few works carried out in vivo, and none in zebrafish embryos despite the advantages related to their utilization. Zebrafish indeed have a set of advantages related to their usefulness in experimental settings, including rapid embryogenesis, high reproductive rate, and genetic similarity to humans [27,28]. This work aims to investigate and report for the first time on the protective effects of resveratrol against ZnO NP-induced toxicity in zebrafish embryos and provide new insight into the underpinning mechanism.

## 2. Results and Discussion

### 2.1. Resveratrol Protects Zebrafish Embryos form ZnO NP-Induced Mortality

**Figure 1:**
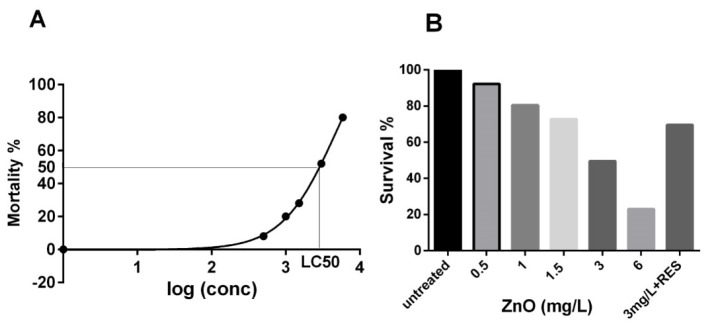
Resveratrol protects zebrafish embryos form ZnO NP-induced mortality. (**A**) log-dose vs. response curve for LC50 determination (see the Materials and Methods for more details). (**B**) The survival rate of the zebrafish embryos treated with different concentrations of ZnO NPs compared to the untreated control group and recovery assay following treatment with 5.0 µM resveratrol (3 mg/L + RES). RES, resveratrol.

To investigate the potential toxicity of ZnO NPs, zebrafish embryos from 24 to 96 h post-fertilization (hpf)were exposed to five different concentrations of NPs (0.5, 1, 1.5, 3, and 6 mg/L), and the percentage of cumulative survival was calculated at 96-hpf. The “no observed effect concentration” (NOEC) was established at 0.5 μg/mL ZnO NPs. Starting from 1.0 μg/mL the lethality score was ≥20% (LOEC—low observed effect concentration), while the lethal concentration required to kill 50% of embryos (LC50) was established at 3 μg/mL (Figure 1A). Resveratrol has been reported to have potentially toxic effects in relation to the concentration employed and the environmental redox state [29]. In this regard, from our dose-response experiments, 5 µM was shown to be the dosage of resveratrol unable to affect the ROS levels in zebrafish embryos.Therefore, to evaluate the protective effect of resveratrol on ZnO NP-induced lethality, we performed a recovery assay, treating embryos from 24 to 96-hpf with 3 μg/mL ZnO NPs (LC50) in the presence/absence of 5 µM resveratrol. The treatment of zebrafish with resveratrol was able to strongly increase the survival rate of 3 mg/L NP-treated embryos (48% survival) as compared to the resveratrol-untreated one (68% survival) reducing the mortality from 52 to 32 percent (Figure 1B). Our results confirm that ZnO NPs are harmful in vivo and provide further evidence for the protective effect of antioxidants toward the toxicity induced by NPs. In this context, our current data represent the first investigation showing the protective role of resveratrol against ZnO NP-induced toxicity in the zebrafish animal model. Notably, human and zebrafish have a high level of genetic orthology, suggesting this small fish may be an important “in vivo model” to study human diseases [27]. Therefore, we believe our findings may provide useful information to translate the use of naturally occurring antioxidants into the treatment, prevention, or amelioration of NP-elicited diseases associated with oxidative stress such as cardiovascular diseases [30,31].

### 2.2. Resveratrol Decreases ZnO NP-Induced Pro-Oxidant Effect

**Figure 2:**
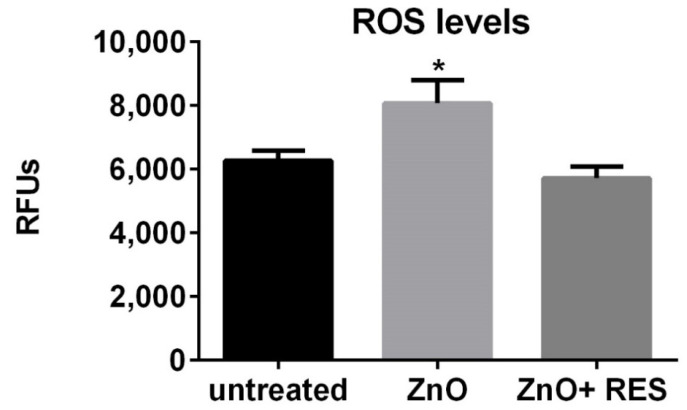
Resveratrol decreases ZnO NP-elicited pro-oxidant effect in zebrafish embryos. Quantitative analysis of ROS level in 72-hpf embryos after exposure to 3 mg/L ZnO NP in the absence (ZnO) or the presence (ZnO + RES) of 5 µM of resveratrol. Fluorescence was measured with a GENios plus microplate reader using a wavelength of 485 nm (excitation) and 535 nm (emission). RES, resveratrol. Values are shown as mean ± SD, *n* = 5; *, significantly different from untreated.

**Figure 3:**
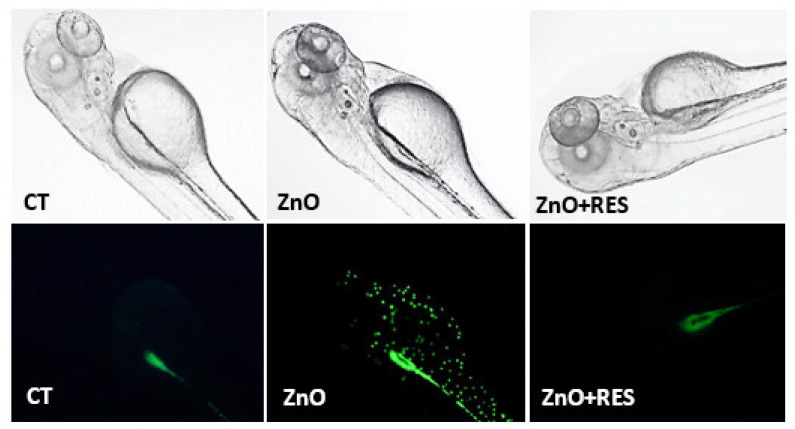
Resveratrol counteracts ZnO NP-induced ROS production in zebrafish embryos. Qualitative localization analysis in 72-hpf zebrafish embryos after staining with 5 μM Carboxy-H2DCFDA probe. (CT) localization of ROS production in untreated zebrafish embryos. (ZnO) localization ROS production in embryos exposed to 3 mg/L ZnO NP. (ZnO + RES) localization ROS production in embryos exposed to 3 mg/L ZnO NP + 5 µM resveratrol. RES, resveratrol. Magnification 10×.

A number of different studies, many of which were conducted in aquatic organisms [16,32], including zebrafish [33,34], demonstrated that oxidative stress is a central mechanism of ZnO NP-induced toxicity. Indeed, in the aquatic environment, Zn^2+^ is partially released from ZnO NPs [16,33] determining, thus, an increased ROS production, which may lead to a redox status imbalance and oxidative stress if not properly counterbalanced by the cellular antioxidant defense. Recent in vitro and in vivo studies show that antioxidants employment can reduce NP-elicited oxidative stress and general toxicity [19,21,22]. However, this field of research is still widely unexplored, since only a few antioxidants have been tested and their potential protective effects, as well as the underpinning mechanisms, are still under active investigation. In this context, resveratrol is a polyphenol produced by several plants in response to injury or pathogen attack [35]. Despite the fact that resveratrol possesses strong antioxidant activity widely documented in several diseases and drug toxicity models associated with oxidative stress [35], its efficacy against ZnO-induced nanotoxicity, as well as the associated molecular mechanisms, remain to be elucidated. In this light, we undertook a set of experiments to provide a new mechanistic insight on the protective effect of resveratrol in the widely used in vivo model, the zebrafish (*Danio rerio*) [27]. We first investigated the ability of resveratrol to prevent ZnO NP-induced oxidative stress in zebrafish embryos. As shown in Figure 2, measurements of ROS levels were significantly higher in the NP-treated larvae than in the control group, confirming the ability of ZnO NPs to induce oxidative stress in zebrafish. Moreover, co-exposure with 5 µM resveratrol strongly decreased the ROS levels and completely counteracted the oxidative stress induced by NPs (Figure 2). Observation of embryos under fluorescence microscopy confirmed the ROS quantification data and revealed the most NP-affected areas in the fish. As compared to the control group (Figure 3, CT), ZnO NP-treated zebrafish larvae showed a higher intensity of DCFDA fluorescence, clearly visible in the abdominal region (Figure 3, ZnO). As further evidence of resveratrol to decrease ROS production, embryos treated with resveratrol showed a markedly reduced fluorescence in the abdominal region (Figure 3, ZnO + RES).

### 2.3. Resveratrol Counteracts ZnO NP-Induced Mitochondrial Damage

**Figure 4:**
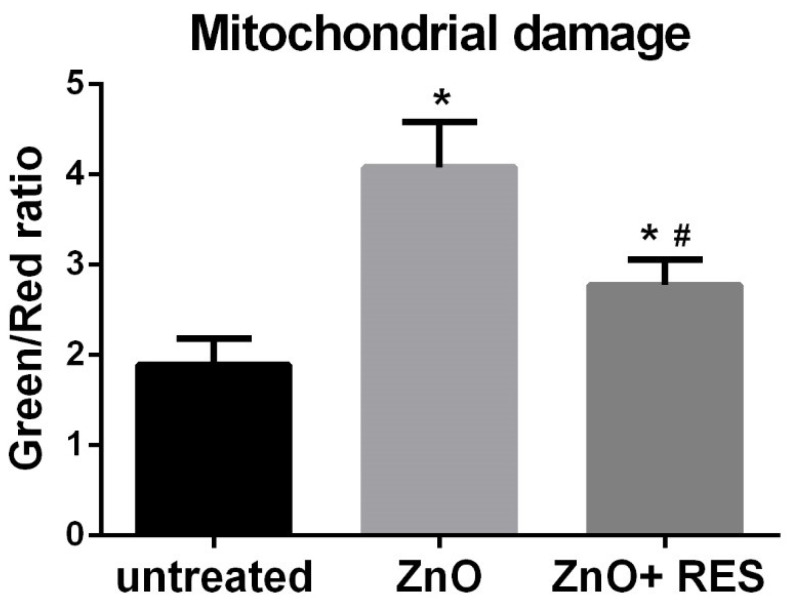
Resveratrol protects mitochondrial function in zebrafish embryos exposed to ZnO NPs. Quantification of mitochondrial damage (green/red fluorescence ratio) in 72-hpf embryos after treatment with 3 mg/L ZnO NP in the absence (ZnO) or presence (ZnO + RES) of 5 μM resveratrol. Embryos were stained with 5 μM JC-1 probe. Fluorescence was measured with a GENios plus microplate reader using a wavelength of 485 nm (excitation) and 535 nm (emission). RES, resveratrol. Values are shown as mean ± SD, *n* = 5. *, significantly different from untreated; #, significantly different from ZnO.

**Figure 5:**
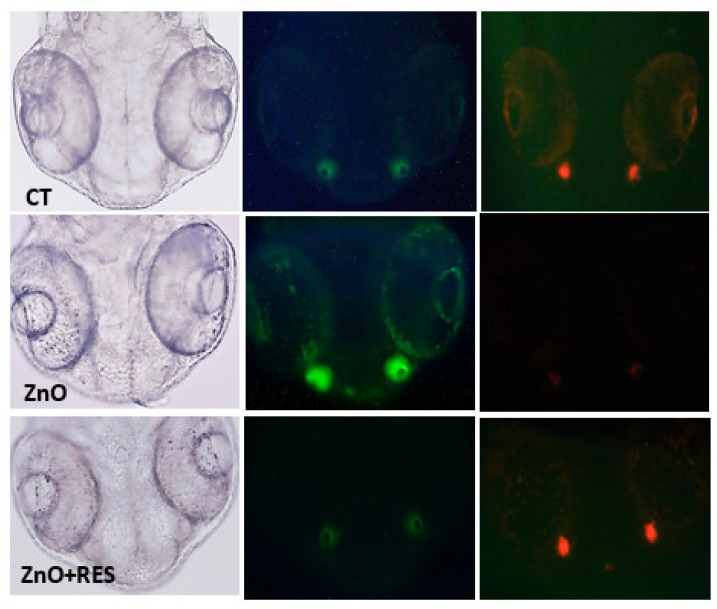
Resveratrol decreases mitochondrial damage induced by ZnO NPs. Qualitative analysis in 72-hpf zebrafish embryos after 5.0 μM JC-1 staining. Fluorescent microscopy images show mitochondrial membrane potential (ΔΨm) in untreated embryos (CT), 3 mg/L ZnO-treated embryos (ZnO NP), and embryos treated with 3 mg/L ZnO in the presence of 5 µM resveratrol (ZnO + RES). Red fluorescence, a sign of preserved ΔΨm, is clearly visible in both non-treated embryos and embryos treated with the addition of RES, whereas embryos treated with ZnO NP display a low red signal, along with a bright green fluorescent signal, index of mitochondrial membrane depolarization. RES, resveratrol. Magnification 20×.

Besides the intrinsic pro-oxidant effect of NPs [36], ROS are also produced in a variety of biochemical reactions within the cell. Mitochondria, for instance, along with their well-known function as a cellular energy (ATP) provider, are the primary source of ROS, as a result of the respiration process [37]. In this regard, mitochondria appear to be the major target of NPs, which, once inside the organelle, can modify mitochondrial functionality (by impairing the electron transport chain or activating the NADPH-like enzyme system) or damage the mitochondrial membrane, resulting in increased release of ROS [38]. NP-induced impairment of mitochondrial functionality has been well documented in human cells [39,40,41]. Recent work in zebrafish show that exposure to ZnO NPs causes a decrease of mitochondrial membrane potential and alters the balance of important membrane proteins (such as Bcl-2 and Ucp-2), their function related to ROS production [34,42]. To understand whether the protective effect exerted by resveratrol in zebrafish may depend on the safeguard of mitochondria function, we measured the mitochondrial membrane potential (ΔΨm) in NP-treated cells both in the absence and presence of resveratrol. For this purpose, the fluorescence JC-1 probe was used to assess mitochondrial function [43,44]. In healthy mitochondria (high membrane potential), JC-1 is incorporated inside the matrix, forming aggregates with bright red fluorescence, while its fluorescence becomes green when it leaves the mitochondria with low membrane potential caused by a damaged mitochondrial membrane. A measure of mitochondrial damage level is, thus, expressed by the green/red fluorescence ratio [43,44,45]. As compared to untreated embryos, zebrafish larvae treated with ZnO NPs showed a marked decrease of membrane potential, indicated by the high green/red ratio value (Figure 4). Alternatively, embryos treated with resveratrol displayed a reduced value of green/red ratio (higher membrane potential), indicating that resveratrol has the ability to reduce the mitochondrial damage elicited by NPs. The mitochondrial protective effect of resveratrol is also clearly underlined in the qualitative imagines captured under a fluorescent microscope (Figure 5), where NP-exposed fish embryos treated with resveratrol (Figure 5, ZnO + Res) showed a red fluorescence as bright as the control group (Figure 5, Ct). On the contrary, embryos treated with only ZnO NPs (ZnO) exhibit a marked shift in fluorescence from red to green, with a red signal scarcely visible.

### 2.4. Resveratrol Prevents ZnO NP-Induced Apoptosis and Necrosis

**Figure 6:**
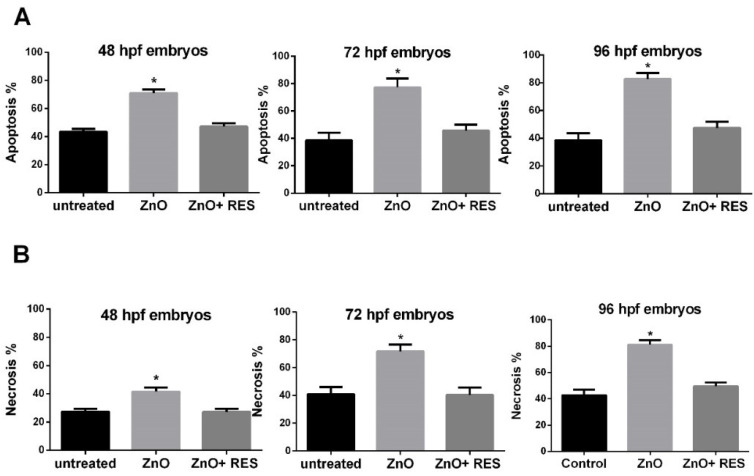
Resveratrol prevents ZnO NP-induced apoptosis and necrosis. (**A**) Percentage of apoptosis and (**B**) necrosis in 48, 72, and 96-hpf zebrafish embryos. Twenty four-hpf embryos were treated with 3 mg/L ZnO in the absence (ZnO) and presence (ZnO + RES) 5 µM resveratrol before proceeding with AO/PI double staining for apoptosis/necrosis quantification. Fluorescence was measured at the indicated hpf with a GENios plus microplate reader using a wavelength of 485 (excitation)/535 (emission) for AO and 493 (excitation)/632 (emission) for PI. RES, resveratrol. Values are shown as mean ± SD, *n* = 5. *, significantly different from untreated.

**Figure 7:**
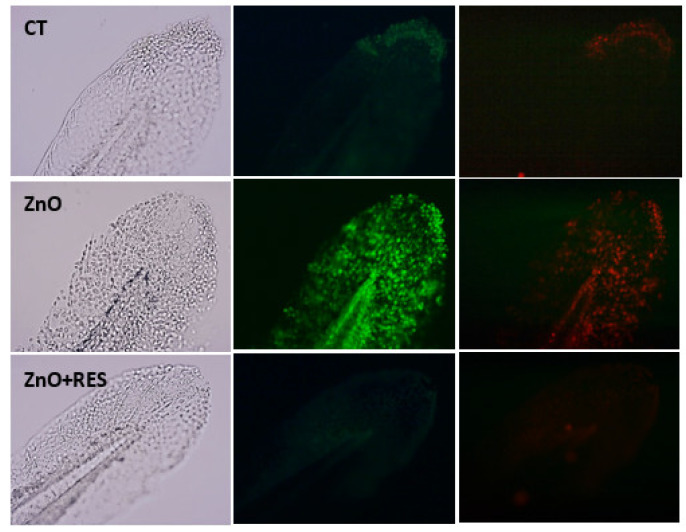
Resveratrol counteracts ZnO NP-induced apoptotic and necrotic fluorescence signals. Fluorescent microscopy images of AO/PI double staining in zebrafish untreated embryos (CT), 3 mg/L ZnO-treated embryos (ZnO NP), and 3 mg/L ZnO + 5 µM resveratrol treated embryos (ZnO + RES). As compared to untreated embryos, ZnO NP-treated zebrafish embryos emit a strong green and red fluorescence signal evidence of increased apoptosis and necrosis, a phenomenon prevented by resveratrol treatment. RES, resveratrol. Magnification 20×.

ROS accumulation causes progressive cellular damage and eventually lead to cell death by apoptosis or/and necrosis [46]. Although sometimes difficult to differentiate, necrosis generally occurs in cases of acute and extensive damage with resulting cell lysis and leakage of cell constituents into the extracellular space [46]. Apoptosis, conversely, is a well-regulated form of cell death, which requires an intact plasma membrane and the action of specific proteases and nucleases [46]. Mitochondrial dysfunction, with loss of membrane permeability and integrity, are typical features of apoptosis [47]. Indeed, mitochondrial damage is generally associated with early apoptosis, whereas DNA damage to a later apoptotic phase [47]. In this regard, mitochondria appear as the main target of NP-induced oxidative stress, and the mitochondrial apoptotic pathway (noted as the intrinsic pathway of apoptosis) plays a prevalent role in cell death induced by the metal oxide NPs [47]. To gain mechanistic insight to ZnO NP-induced cell damage in zebrafish, the acridine orange/propidium iodide (AO/PI) assay was carried out to evaluate the prevalence of apoptosis and necrosis in fish embryos treated with ZnO-NP. Quantitative results depicted in Figure 6A,B indicated a high level of both apoptosis (Figure 6A) and necrosis (Figure 6B), providing evidence of ZnO NP ability to induce in vivo damage of zebrafish embryos. The same picture indicates that the NP-induced cell impairment was completely prevented by resveratrol treatment, confirming resveratrol protection against ZnO NP-induced cell damage, and further highlighting that antioxidants may be successfully employed to reduce NPs toxicity (Figure 6A,B). The double staining AO/PI image offers the advantage to potentially discriminate between apoptotic cells, with the intact membrane, and necrotic cells with the damaged membrane. Indeed, apoptotic cells show a very bright green signal because of AO binding to the condensed chromatin in the nucleus, while necrotic dead cells with damaged membrane display a red fluorescence signal due to PI accumulation inside the cytoplasm. Qualitative microscope analysis of zebrafish embryos stained with AO/PI paralleled quantitative data, clearly displaying an increase of both green (apoptosis) and red fluorescence (necrosis) following ZnO NPs treatment, an effect dramatically counteracted by the resveratrol which reported both fluorescence levels comparable to the untreated fish (Figure 7). Again, the current data represent the first evidence regarding the protective effect of resveratrol against ZnO NP-induced toxicity in zebrafish. 

### 2.5. Resveratrol Counteracts Cardiac Vascular Modifications and Function Induced by ZnO NP

**Figure 8:**
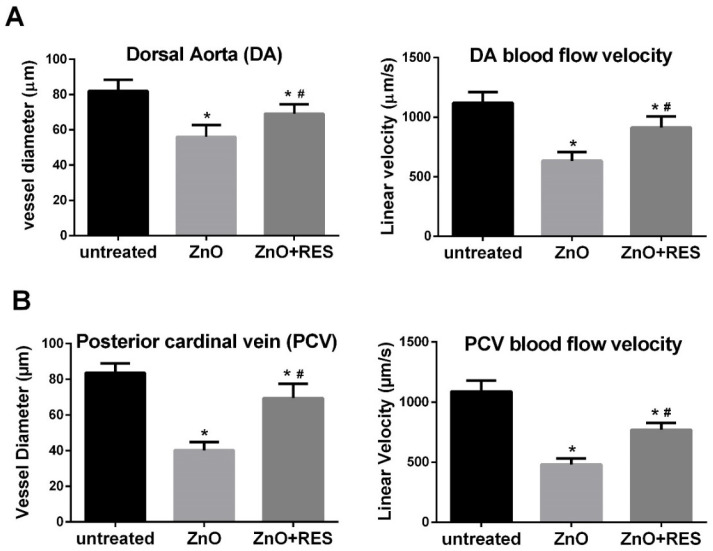
Resveratrol ameliorates cardiac vascular modifications and function induced by ZnO NP. Vessel diameter and blood flow velocity in the two major blood vessels (**A**) dorsal aorta (DA) and (**B**) posterior cardinal vein (PVC) was measured in 96-hpf zebrafish embryos treated with 3 mg/L ZnO NP w/wo 5.0 µM resveratrol. RES, resveratrol. Values are shown as mean ± SD, *n* = 5. *, significantly different from untreated; #, significantly different from ZnO.

Next, we investigated the ability of resveratrol to reduce zebrafish fetal malformations induced by ZnO NPs. To this end, zebrafish embryos were examined after three days of NPs treatment in both the presence and absence of the antioxidant polyphenol. For each individual embryo, typical developmental-associated phenotypes such as body length, eye size, yolk size, and cardiac functions like heartbeat, the diameter of first blood vessels, and blood flow velocity were assessed. Images were captured by the HCImage software, version 4.4.1.0 (Hamamatsu Photonics, Tsukuba, Japan) and analyzed with the ImageJ software version 1.52a. No standard body measurements (body length, eye size, yolk size) variation was detected in embryos exposed to ZnO NPs for three days, except for the eyes size that presented larger as compared to the control group. On the contrary, cardiovascular functions instead showed significant modifications in response to the embryos exposition to ZnO NP (Figure 8A,B). In particular, the diameter of both dorsal aorta (DA) and posterior cardinal vein (PCV) was strongly reduced in NP-treated embryos, a phenomenon that was significantly ameliorated by resveratrol treatment (Figure 8A,B). Moreover, blood flow velocity in both DA and PVC was significantly affected in ZnO NP-treated fish, but once again, resveratrol was able to significantly counteract the NP-induced effect (Figure 8A,B). ZnO NPs exposure had no significant effect on the heartbeat in the analyzed zebrafish embryos. 

### 2.6. Resveratrol Ameliorates Morphological Vascular Modifications Induced by ZnO NPs

**Figure 9:**
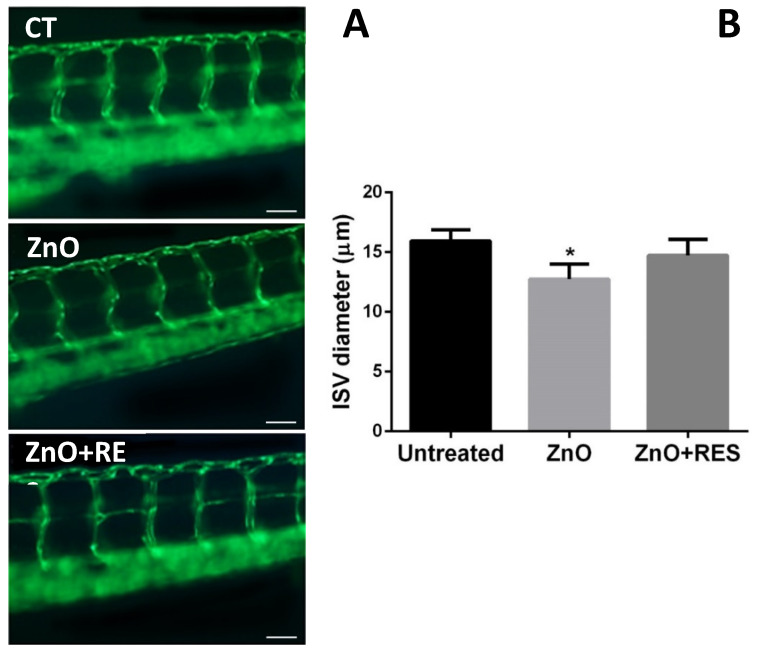
Resveratrol ameliorates alterations in the width of intersegmental vessels. (**A**) Representative fluorescence pictures depicting intersegmental vessels (ISVs) in the 96-hpf Fli1-GFP embryos exposed to 3 mg/L ZnO-NPs (ZnO) indicting a reduced vessel width as compared to the dimension of the control group (CT). Embryos exposed to 3 mg/L ZnO + 5 µM resveratrol (ZnO + RES) display a decrease in the incidence of modified vessels, which appear both regular and with reduced NP-induced phenotype modification. Scale Bar 50 µM. (**B**) Quantification of vessel diameter was performed using ImageJ (v1.44 public domain software http://rsbweb.nih.gov/ij/) in 96 hpf embryos. RES, resveratrol. Values are shown as mean ± SD, *n* = 20. *, significantly different from the untreated. (Magnification 20×).

**Figure 10:**
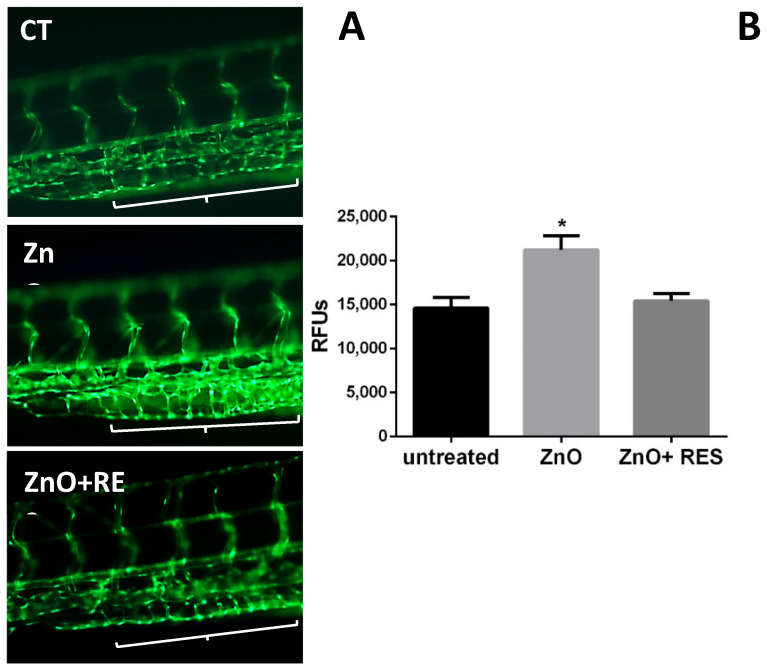
Resveratrol decreases the thickness of the blood vessel in ZnO NP-treated embryos. (**A**) Vessel of caudal vein plexus region of 96-hpf Fli1 embryos treated with 3 mg/L ZnO NP (ZnO) showed a significant increase in green fluorescence intensity (Thickening) compare to the untreated group (CT). Treatment of 5 µM resveratrol resulted in the prevention of NP-induced GFP signal increment (ZnO + RES). Regions of interest are marked by a white bracket. (**B**) Quantification of vessel thickness intensity was performed using ImageJ (v1.44 public domain software http://rsbweb.nih.gov/ij/) in 96-hpf embryos. RES, resveratrol. Values are shown as mean ± SD, *n* = 20. *, significantly different from the untreated. Magnification 20×.

Alterations in blood flow during embryonal development are often associated with heart defects in the adult stage [48]. Moreover, recent work in zebrafish reported that a reduction of blood flow and vessel diameters in ZnO NP- and ceramide-treated embryos is related to abnormal blood vessel development [49,50]. The primitive circulatory system in zebrafish consists of the dorsal aorta and cardinal vein [51]. At one-day post-fertilization, angiogenesis occurs in the trunk leading to the formation of intersegmental vessels (ISVs) [51]. These vessels, due to their typical “S-like” phenotype, with a wavy structure and a constant width, represent a valid target for investigating angiogenic alterations [52,53]. In this context, we next investigated whether resveratrol protection against ZnO NP-induced damage may extend to the zebrafish vasculature. To better address this question and have a clearer image of the zebrafish vasculature, we used the transgenic zebrafish line (Flil:EGFP), where fish express an enhanced green fluorescence protein (EGFP) under the control of the endothelial-specific promoter (fli1) [54]. Exposure to ZnO-NPs resulted in the production of two main modified phenotypes in fli1 embryos of 96 and 120 hpf (Figure 9 and Figure 10). Intersegmental vessels of ZnO NP-treated zebrafish, although showing a regular wavy pattern, displayed an altered width significantly smaller as compared to that of the untreated group (Figure 9). As reported in the same image, the NP ZnO-elicited phenotype was prevented by the addition of resveratrol (Figure 9), resulting in embryos showing vessel patterns similar to that of untreated fish (Figure 9). ZnO NP-vasotoxicity on zebrafish was further confirmed by causing a modification of the vessel thickening in the caudal vein plexus region (Figure 10). As reported both qualitatively and quantitatively in Figure 10, NP-induced vessel thickening appeared as more intense EGFP fluorescence, which was efficiently and significantly attenuated by resveratrol treatment. The current findings on vascular phenotypes modification, besides representing additional evidence of the potential harm ZnO NP’s have toward the vascular system, highlight once again the ability of resveratrol to blunt the ZnO NP-exerted effects and the beneficial action of antioxidants in counteracting or preventing cardiovascular diseases associated to NPs exposure.

## 3. Materials and Methods

### 3.1. Chemicals

ZnO NPs dispersion 50 WT% in H2O, particle size ≤ 100 nm were purchased from Sigma–Aldrich, MO, USA (Catalog n° 721077). E3 medium (egg water) with N-phenylthiourea (PTU) (Sigma, Steinheim, Germany) used either to cultivate zebrafish embryos and to inhibit pigment formation during embryos development and facilitate their visualization under the microscope. Constituents of E3 media are: 5.0 mM sodium NaCl, 0.17 mM KCl, 0.33 mM MgSO_4_∙7H_2_O and 0.33 mM CaCl_2_∙2H_2_O, all purchased from Sigma, Steinheim, Germany. To prevent fungal, bacterial, and viral contamination, Methylene blue (Kordon-USA) was added in the E3 medium. ROS and mitochondrial membrane potential measurements were performed using respectively Carboxy-H2DCFDA probe (#C400-Thermo Fisher, MA, USA) and JC-1 (#T3168 Thermo Fisher, MA, USA). Apoptosis and necrosis were determined using Acridine Orange (AO) hydrochloride solution 10 mg/mL in H2O (#A8097 Sigma–Aldrich, MO, USA) and propidium iodide (PI), stock solution 1 mg/mL in H2O (# P4170. Sigma–Aldrich, MO, USA) respectively. Resveratrol (# R5010 Sigma–Aldrich, MO, USA) stock solution 50 mM in Ethanol. Stock solutions for zebrafish embryos experiments, including phosphate buffer saline (PBS) PTU, E3 medium, and methylene blue solution, were prepared as previously described [55,56].

### 3.2. NPs Suspension Preparation

Stock suspension of nano-ZnO (50 mg/L in E3 medium) was prepared fresh and dispersed using a probe sonicator (Fisherbrand™ Q55 Sonicator. Fisher Scientific, Waltham, MA, USA) in an ice bath, for 30 s and 50 amplitude. All used treatments were dispersed in E3 medium and sonicated with the same conditions described above. This type ZnO NPs have been widely employed and prepared suspensions characterized [26,57,58,59].

### 3.3. Zebrafish Husbandry

All the study experimentations, including zebrafish culture, breeding, embryos collection, embryonic and larval culture were performed according to the local and international regulations. All the assays complied with animal protocol guidelines required by the Qatar University Institutional Animal Care and Use Committee (IACUC) and Policy on Zebrafish Research established by the Department of Research in the Ministry of Public Health, Qatar. Wild-type AB and transgenic zebrafish TG (flil:EGFP) zebrafish (*Danio rerio*) lines were used in the toxicity experiments. The AB strain was primarily obtained in 2014 from the Model Fish Facility (MFU), Norwegian University of Life Sciences, Department of Production Animal Clinical Sciences, Oslo, Norway. Zebrafish were maintained in an environmentally controlled lab (14 h light/10 h dark cycle with a water temperature of 28 °C) [60,61] using the Aquaneering system (San Diego, CA, USA) in the Biomedical Research Center (BRC) at Qatar University (QU). Brine shrimps and dry food was provided to the adult fish twice every day. The levels of conductivity, temperature, and pH were monitored continuously and simultaneously by the sensors of the semi-automated aquaneering system. Zebrafish were prepared for mating by locating two pairs of adult female and male fish in a single mating tank separated by a divider and left in the dark overnight. The next morning, spawning was triggered by removing the divider. The embryos were left to mate for 5 h, then healthy fertilized eggs were collected and washed with PTU-E3 media before conducting the experiments.

### 3.4. Acute Toxicity Assays

To evaluate the toxic effect of ZnO NPs, a dose-response assay was performed to determine the median lethal dose (LC50). Zebrafish embryos (25 for each concentration) were treated for 3 days, from 24 to 96 hpf, with five different concentrations (0.5, 1, 1.5, 3, and 6 mg/L) of ZnO NPs dispersion. Embryos were distributed in 12-well plates and counted for lethality every 24 h. Mortality rates were recorded using the Ziess Stemi 2000-C stereomicroscope by observation of heart beating, motility and color of tissues that change to opaque in dead embryos. A sigmoidal curve was graphed to show the dose-inhibition response, in which the percentage of mortality represents the y-axis and the Log (Concentration) in the x-axis [62]. Then, the median lethal dose (LC50) was determined from the curve using the Graph Pad Prism Software [63]. In addition, no observed effect concentration (NOEC) and low observed effect concentration (LOEC) were estimated. NOEC is the concentration at which the mortality score is <20%, whereas LOEC is when the death rate is equal to or >20%.

### 3.5. Zebrafish Embryo Imaging and Cardiotoxicity Assay

Imaging of zebrafish embryos was performed with Zeiss SteREO Discovery V8 Microscope equipped with Hamamatsu Orca Flash high-speed camera and HCImage software, version 4.4.1.0 (Hamamatsu Photonics, Tsukuba, Japan) as described in [64]. 96-hpf selected embryos were placed on a depression slide with 1–2 drops of 3% methylcellulose. Embryos were oriented and positioned on their side for measurement of the two major blood vessels, dorsal aorta (DA) and posterior cardinal vein (PCV). The captured images were analyzed using ImageJ software version 1.52a (NIH, Washington DC, USA) bundled with Java 1.8.0 in which variations in the size of the yolk, eye, and body length were measured. Moreover, videos of the tail were recorded for all embryos at the same site where the two major blood vessels were visible. Then, MicroZebraLab Blood Flow software from ViewPoint (Lyon, France) was used to measure three parameters; blood flow velocity, vessel diameter, and heart rate for both blood vessels [64].

### 3.6. ROS Measurement

ROS levels were determined by using the ROS fluorescent probe Carboxy-H2DCFDA. The non-fluorescent probe molecule diffuses into cells where once deacetylated by cellular esterase, is converted into a highly fluorescent green inform presence in the presence of ROS. Zebrafish embryos (72 hpf) were first incubated with 5.0 μM of the probe for 60 min, in the dark, and then washed three times with E3 medium before exposure to the selected concentrations of ZnO NPs dispersion, with or without 5 µM resveratrol. Before proceeding to further analysis (imaging and quantitative assessment), embryos were anesthetized with tricaine. Fluorescence intensity was measured with GENios plus microplate reader (Tecan, Männedorf, CH) at the excitation wavelength of 485 nm and an emission wavelength of 535 nm, using a 96-multiwell dark plate with 5/6 embryos per well. Results were normalized to background fluorescence, in both wells containing only solution and wells containing zebrafish embryos untreated with ROS probe. Data are representative of five independent experiments with a total number of 50/60 embryos for each experiment and were expressed as a means ± SD of the relative fluorescence unit (RFU) values. Imaging and qualitative ROS evaluation were carried out by observation of live embryos under a fluorescence microscope Zeiss (SteREO Discovery V8 Microscope) [65,66,67,68,69].

### 3.7. Mitochondrial Membrane Potential (ΔΨm) Measurement

JC-1 is a cationic dye that accumulates in mitochondria in a membrane potential-dependent manner. At low membrane potentials, JC-1 exists in monomeric form and emits green fluorescent (490 nm excitation/530 nm emission), whereas at high membrane potential is in an aggregated form and exhibits a red fluorescent (530 nm excitation/590 nm emission). A decrease in membrane potential is, therefore, indicated by a fluorescence emission shift from green to red. A 1.5 mM stock solution in DMSO was prepared according to the manufacturer’s instructions. 72-hpf zebrafish embryos were incubated in the dark for 30 min with 5.0 μM JC-1 solution in PBS. After incubation time, embryos were washed three times with E3 medium and then treated with selected concentrations of ZnO NPs, with or without resveratrol. Fluorescence intensity was measured with GENios plus microplate reader (Tecan, Männedorf, CH) with both red (535–590 nm) and green (485–535 nm) channels, using a 96-multiwell dark plate with 5/6 embryos per well. The images were captured using a fluorescent microscope with red and green channels. Both qualitative and qualitative analyses were performed in vivo with tricaine-anesthetized embryos. Data (representative of 5 independent experiments with a total number of 50/60 embryos for each experiment) are expressed as a means ± SD of the relative fluorescence unit (RFU) values and have been normalized to background fluorescence (wells containing only solution and wells containing zebrafish embryos untreated with JC-1 probe). The results expressed as the ratio of average red/green fluorescence signals represent quantification of the degree of Δψm level [43,44,45].

### 3.8. Apoptosis and Necrosis Determination

Apoptosis and necrosis were detected by dual staining with two fluorochromes, acridine orange (AO) and propidium iodide (PI), both able to intercalate DNA and to emit green and red fluorescence, respectively. Double staining AO/PI offers the advantage of discriminating between live, apoptotic, and dead cells. AO is membrane permeable and can only stain viable cells, which appear with a green nucleus, and early apoptotic cells, showing a bright-green signal due to chromatin condensation. Propidium iodide can cross-cells, which have lost their membrane integrity (late apoptotic and necrotic cells) and emit red fluorescence. It is worth to be mentioned that AO can intercalate both dsDNA and ssDNA or RNA. When it is bound to dsDNA, emit a green fluorescence, but when it intercalates ssDNA or RNA, the fluorescence signal is red. However, with AO/PI double staining, only dead nucleated cells fluoresce red and live nucleated cells in green. This is due to the Förster resonance energy transfer (FRET) phenomenon for which the PI signal absorbs the AO signal, without overflow or double-positive results. Twenty four-hpf zebrafish embryos were used for treatment with selected concentrations of ZnO NPs dispersion, with or without resveratrol. For apoptosis/necrosis analysis by AO/PI double staining 48, 72, and 96-hpf Zebrafish embryos were incubated with 10 μg/mL AO and 10 μg/mL PI for 15 min, in the dark, then they were washed three times with E3 medium. A quantitative analysis was performed measuring fluorescence intensity with GENios plus microplate reader (Tecan, Männedorf, CH) with 485 excitation/535 emissions for AO and 493 excitation/f632 emission for PI. A total of 50 embryos (five per well) were used in a 96-multiwell dark plate. The results are representative of three independent experiments and are expressed as a means ± SD of the relative fluorescence unit (RFU) values. Data are normalized to background fluorescence (wells containing only solution and wells containing unstained zebrafish). Imaging analysis was carried out with a fluorescent microscope with both red and green channels using live embryos anesthetized with tricaine [58,70].

### 3.9. Statistical Analysis

Data are expressed as means ± S.D. of three or five different experiments. One-way analysis of variance (ANOVA) followed by a posthoc Newman–Keuls Multiple Comparison Test was used to detect differences of means among treatments with significance defined as *p* < 0.05. Statistical analysis was performed using GraphPad Prism version 5.00 for Windows (GraphPad Software, San Diego, CA, USA).

## 4. Conclusions

In the present work, we provided evidence that resveratrol can reduce ZnO NP-induced zebrafish lethality and prevent damage during the embryos’ development and vascularization. Mechanistically, resveratrol was able to inhibit oxidative stress, prevent mitochondrial dysfunction, and counteract cell apoptosis/necrosis elicited by ZnO NP. We are aware that the identification of the fine molecular mechanism of ZnO NP-induced cell damage requires further investigations. For instance, whether resveratrol protection and antioxidant effects against ZnO NPs are due to its direct scavenger action or activation of antioxidant defense mechanisms remains to be elucidated and will be the goal of our future investigation. Nevertheless, this work provides further evidence concerning the primary role of oxidative stress in NPs induced damage, and a potential role that antioxidants may exert in counteracting nanomaterial toxicity.

## References

[1] Hasan S. (2015). A review on nanoparticles: Their synthesis and types. Res. J. Recent Sci..

[2] Christian P., Von der Kammer F., Baalousha M., Hofmann T. (2008). Nanoparticles: Structure, properties, preparation and behaviour in environmental media. Ecotoxicology.

[3] Khan I., Saeed K., Khan I. (2019). Nanoparticles: Properties, applications and toxicities. Arab. J. Chem..

[4] Osmond M.J., McCall M.J. (2010). Zinc oxide nanoparticles in modern sunscreens: An analysis of potential exposure and hazard. Nanotoxicology.

[5] Bondarenko O., Juganson K., Ivask A., Kasemets K., Mortimer M., Kahru A. (2013). Toxicity of Ag, CuO and ZnO nanoparticles to selected environmentally relevant test organisms and mammalian cells in vitro: A critical review. Arch. Toxicol..

[6] Vandebriel R.J., De Jong W.H. (2012). A review of mammalian toxicity of ZnO nanoparticles. Nanotechnol. Sci. Appl..

[7] Bai L., Shin S., Burnett R.T., Kwong J.C., Hystad P., van Donkelaar A., Goldberg M.S., Lavigne E., Copes R., Martin R.V. (2019). Exposure to ambient air pollution and the incidence of congestive heart failure and acute myocardial infarction: A population-based study of 5.1 million Canadian adults living in Ontario. Environ. Int..

[8] Scherzad A., Meyer T., Kleinsasser N., Hackenberg S. (2017). Molecular mechanisms of Zinc Oxide nanoparticle-induced genotoxicity short running title: Genotoxicity of ZnO NPs. Materials.

[9] Singh S. (2019). Zinc oxide nanoparticles impacts: Cytotoxicity, genotoxicity, developmental toxicity, and neurotoxicity. Toxicol. Mech. Methods.

[10] Fu P.P., Xia Q., Hwang H.M., Ray P.C., Yu H. (2014). Mechanisms of nanotoxicity: Generation of reactive oxygen species. J. Food Drug Anal..

[11] Yang Y., Bazhin A.V., Werner J., Karakhanova S. (2013). Reactive oxygen species in the immune system. Int. Rev. Immunol..

[12] Park E.J., Park K. (2009). Oxidative stress and pro-inflammatory responses induced by silica nanoparticles in vivo and in vitro. Toxicol. Lett..

[13] Limon-Pacheco J., Gonsebatt M.E. (2009). The role of antioxidants and antioxidant-related enzymes in protective responses to environmentally induced oxidative stress. Mutat. Res..

[14] Khanna P., Ong C., Bay B.H., Baeg G.H. (2015). Nanotoxicity: An interplay of oxidative stress, inflammation and cell death. Nanomaterials.

[15] Saddick S., Afifi M., Abu Zinada O.A. (2017). Effect of Zinc nanoparticles on oxidative stress-related genes and antioxidant enzymes activity in the brain of Oreochromis niloticus and Tilapia zillii. Saudi J. Biol. Sci..

[16] Kaya H., Aydin F., Gurkan M., Yilmaz S., Ates M., Demir V., Arslan Z. (2015). Effects of zinc oxide nanoparticles on bioaccumulation and oxidative stress in different organs of tilapia (*Oreochromis niloticus*). Environ. Toxicol. Pharmacol..

[17] Attia H., Nounou H., Shalaby M. (2018). Zinc oxide nanoparticles induced oxidative dna damage, inflammation and apoptosis in rat’s brain after oral exposure. Toxics.

[18] Alkaladi A. (2019). Vitamins E and C ameliorate the oxidative stresses induced by zinc oxide nanoparticles on liver and gills of Oreochromis niloticus. Saudi J. Biol. Sci..

[19] Fukui H., Iwahashi H., Endoh S., Nishio K., Yoshida Y., Hagihara Y., Horie M. (2015). Ascorbic acid attenuates acute pulmonary oxidative stress and inflammation caused by zinc oxide nanoparticles. J. Occup. Health.

[20] Sarkar A., Sil P.C. (2014). Iron oxide nanoparticles mediated cytotoxicity via PI3K/AKT pathway: Role of quercetin. Food Chem. Toxicol..

[21] Gonzalez-Esquivel A.E., Charles-Nino C.L., Pacheco-Moises F.P., Ortiz G.G., Jaramillo-Juarez F., Rincon-Sanchez A.R. (2015). Beneficial effects of quercetin on oxidative stress in liver and kidney induced by titanium dioxide (TiO_2_) nanoparticles in rats. Toxicol. Mech. Methods.

[22] Sonane M., Moin N., Satish A. (2017). The role of antioxidants in attenuation of Caenorhabditis elegans lethality on exposure to TiO_2_ and ZnO nanoparticles. Chemosphere.

[23] Gülçin İ. (2010). Antioxidant properties of resveratrol: A structure–activity insight. Innov. Food Sci. Emerg. Technol..

[24] Hu J., Zhang B., Du L., Chen J., Lu Q. (2017). Resveratrol ameliorates cadmium induced renal oxidative damage and inflammation. Int. J. Clin. Exp. Med..

[25] Emsen B., Turkez H. (2017). The protective role of resveratrol against zinc oxide induced nanotoxicity. Anatol. J. Bot..

[26] Abdel-Daim M.M., Eissa I.A.M., Abdeen A., Abdel-Latif H.M.R., Ismail M., Dawood M.A.O., Hassan A.M. (2019). Lycopene and resveratrol ameliorate zinc oxide nanoparticles-induced oxidative stress in Nile tilapia, Oreochromis niloticus. Environ. Toxicol. Pharmacol..

[27] Lieschke G.J., Currie P.D. (2007). Animal models of human disease: Zebrafish swim into view. Nat. Rev. Gen..

[28] Nasrallah G.K., Zhang Y., Zagho M.M., Ismail H.M., Al-Khalaf A.A., Prieto R.M., Albinali K.E., Elzatahry A.A., Deng Y. (2018). A systematic investigation of the bio-toxicity of core-shell magnetic mesoporous silica microspheres using zebrafish model. Microporous Mesoporous Mater..

[29] Shaito A., Posadino A.M., Younes N., Hasan H., Halabi S., Alhababi D., Al-Mohannadi A., Abdel-Rahman W.M., Eid A.H., Nasrallah G.K. (2020). Potential Adverse Effects of Resveratrol: A Literature Review. Int. J. Mol. Sci..

[30] Cao Y., Gong Y., Liao W., Luo Y., Wu C., Wang M., Yang Q. (2018). A review of cardiovascular toxicity of TiO_2_, ZnO and Ag nanoparticles (NPs). Biometals.

[31] Aldosari S., Awad M., Harrington E.O., Sellke F.W., Abid M.R. (2018). Subcellular Reactive Oxygen Species (ROS) in Cardiovascular Pathophysiology. Antioxidants.

[32] Hao L., Chen L. (2012). Oxidative stress responses in different organs of carp (*Cyprinus carpio*) with exposure to ZnO nanoparticles. Ecotoxicol. Environ. Saf..

[33] Xiong D., Fang T., Yu L., Sima X., Zhu W. (2011). Effects of nano-scale TiO_2_, ZnO and their bulk counterparts on zebrafish: Acute toxicity, oxidative stress and oxidative damage. Sci. Total Environ..

[34] Zhao X., Wang S., Wu Y., You H., Lv L. (2013). Acute ZnO nanoparticles exposure induces developmental toxicity, oxidative stress and DNA damage in embryo-larval zebrafish. Aquat. Toxicol..

[35] Repossi G., Das U.N., Eynard A.R. (2020). Molecular Basis of the Beneficial Actions of Resveratrol. Arch. Med. Res..

[36] Meng H., Xia T., George S., Nel A.E. (2009). A predictive toxicological paradigm for the safety assessment of nanomaterials. ACS Nano.

[37] Marchi S., Giorgi C., Suski J.M., Agnoletto C., Bononi A., Bonora M., De Marchi E., Missiroli S., Patergnani S., Poletti F. (2012). Mitochondria-ros crosstalk in the control of cell death and aging. J. Signal. Transduct..

[38] Chen L., Wu L.Y., Yang W.X. (2018). Nanoparticles induce apoptosis via mediating diverse cellular pathways. Nanomedicine.

[39] Sharma V., Anderson D., Dhawan A. (2012). Zinc oxide nanoparticles induce oxidative DNA damage and ROS-triggered mitochondria mediated apoptosis in human liver cells (HepG2). Apoptosis.

[40] Wang S.-W., Lee C.-H., Lin M.-S., Chi C.-W., Chen Y.-J., Wang G.-S., Liao K.-W., Chiu L.-P., Wu S.-H., Huang D.-M. (2020). ZnO Nanoparticles Induced Caspase-Dependent Apoptosis in Gingival Squamous Cell Carcinoma through Mitochondrial Dysfunction and p70S6K Signaling Pathway. Int. J. Mol. Sci..

[41] Vallabani N.V.S., Sengupta S., Shukla R.K., Kumar A. (2019). ZnO nanoparticles-associated mitochondrial stress-induced apoptosis and G2/M arrest in HaCaT cells: A mechanistic approach. Mutagenesis.

[42] Zhao X., Ren X., Zhu R., Luo Z., Ren B. (2016). Zinc oxide nanoparticles induce oxidative DNA damage and ROS-triggered mitochondria-mediated apoptosis in zebrafish embryos. Aquat. Toxicol..

[43] Posadino A.M., Cossu A., Giordo R., Zinellu A., Sotgia S., Vardeu A., Hoa P.T., Nguyen le H.V., Carru C., Pintus G. (2015). Resveratrol alters human endothelial cells redox state and causes mitochondrial-dependent cell death. Food Chem. Toxicol..

[44] Posadino A.M., Cossu A., Giordo R., Zinellu A., Sotgia S., Vardeu A., Hoa P.T., Deiana L., Carru C., Pintus G. (2013). Coumaric acid induces mitochondrial damage and oxidative-mediated cell death of human endothelial cells. Cardiovasc. Toxicol..

[45] Cossu A., Posadino A.M., Giordo R., Emanueli C., Sanguinetti A.M., Piscopo A., Poiana M., Capobianco G., Piga A., Pintus G. (2012). Apricot melanoidins prevent oxidative endothelial cell death by counteracting mitochondrial oxidation and membrane depolarization. PLoS ONE.

[46] Fink S.L., Cookson B.T. (2005). Apoptosis, pyroptosis, and necrosis: Mechanistic description of dead and dying eukaryotic cells. Infect. Immun..

[47] Ryter S.W., Kim H.P., Hoetzel A., Park J.W., Nakahira K., Wang X., Choi A.M. (2007). Mechanisms of cell death in oxidative stress. Antioxid. Redox Signal..

[48] Hove J.R., Koster R.W., Forouhar A.S., Acevedo-Bolton G., Fraser S.E., Gharib M. (2003). Intracardiac fluid forces are an essential epigenetic factor for embryonic cardiogenesis. Nature.

[49] Kteeba S.M., El-Ghobashy A.E., El-Adawi H.I., El-Rayis O.A., Sreevidya V.S., Guo L., Svoboda K.R. (2018). Exposure to ZnO nanoparticles alters neuronal and vascular development in zebrafish: Acute and transgenerational effects mitigated with dissolved organic matter. Environ. Pollut..

[50] Bansode R.R., Ahmedna M., Svoboda K.R., Losso J.N. (2011). Coupling in vitro and in vivo paradigm reveals a dose dependent inhibition of angiogenesis followed by initiation of autophagy by C6-ceramide. Int. J. Biol. Sci..

[51] Isogai S., Horiguchi M., Weinstein B.M. (2001). The vascular anatomy of the developing zebrafish: An atlas of embryonic and early larval development. Dev. Biol..

[52] Tobia C., Gariano G., Guerra J., Presta M. (2015). Zebrafish embryo intersegmental vessels: A tool for investigating sprouting angiogenesis. Methods Mol. Biol..

[53] Chappell J.C., Wiley D.M., Bautch V.L. (2012). How blood vessel networks are made and measured. Cells Tissues Organs.

[54] Delov V., Muth-Köhne E., Schäfers C., Fenske M. (2014). Transgenic fluorescent zebrafish Tg (fli1: EGFP) y1 for the identification of vasotoxicity within the zFET. Aquat. Toxicol..

[55] Younes N., Salem R., Al-Asmakh M., Altamash T., Pintus G., Khraisheh M., Nasrallah G.K. (2018). Toxicity evaluation of selected ionic liquid compounds on embryonic development of Zebrafish. Ecotoxicol. Environ. Saf..

[56] Nasrallah G.K., Salem R., Da’as S., Al-Jamal O.L.A., Scott M., Mustafa I. (2019). Biocompatibility and toxicity of novel iron chelator Starch-Deferoxamine (S-DFO) compared to zinc oxide nanoparticles to zebrafish embryo: An oxidative stress based apoptosis, physicochemical and neurological study profile. Neurotoxicol. Teratol..

[57] Pranjali P., Meher M.K., Raj R., Prasad N., Poluri K.M., Kumar D., Guleria A. (2019). Physicochemical and Antibacterial Properties of PEGylated Zinc Oxide Nanoparticles Dispersed in Peritoneal Dialysis Fluid. ACS Omega.

[58] Ng C.T., Yong L.Q., Hande M.P., Ong C.N., Yu L.E., Bay B.H., Baeg G.H. (2017). Zinc oxide nanoparticles exhibit cytotoxicity and genotoxicity through oxidative stress responses in human lung fibroblasts and Drosophila melanogaster. Int. J. Nanomed..

[59] Sarkheil M., Johari S.A., An H.J., Asghari S., Park H.S., Sohn E.K., Yu I.J. (2018). Acute toxicity, uptake, and elimination of zinc oxide nanoparticles (ZnO NPs) using saltwater microcrustacean, Artemia franciscana. Environ. Toxicol. Pharmacol..

[60] Korenbrot J.I., Mehta M., Tserentsoodol N., Postlethwait J.H., Rebrik T.I. (2013). EML1 (CNG-modulin) controls light sensitivity in darkness and under continuous illumination in zebrafish retinal cone photoreceptors. J. Neurosci..

[61] Abou-Saleh H., Younes N., Rasool K., Younis M.H., Prieto R.M., Yassine H.M., Mahmoud K.A., Pintus G., Nasrallah G.K. (2019). Impaired Liver Size and Compromised Neurobehavioral Activity are Elicited by Chitosan Nanoparticles in the Zebrafish Embryo Model. Nanomaterials.

[62] Younes N., Pintus G., Al-Asmakh M., Rasool K., Younes S., Calzolari S., Mahmoud K.A., Nasrallah G.K. (2019). “Safe” chitosan/zinc oxide nanocomposite has minimal organ-specific toxicity in early stages of zebrafish development. ACS Biomater. Sci. Eng..

[63] Hussein E.A., Zagho M.M., Rizeq B.R., Younes N.N., Pintus G., Mahmoud K.A., Nasrallah G.K., Elzatahry A.A. (2019). Plasmonic MXene-based nanocomposites exhibiting photothermal therapeutic effects with lower acute toxicity than pure MXene. Int. J. Nanomed..

[64] Al-Kandari H., Younes N., Al-Jamal O., Zakaria Z.Z., Najjar H., Alserr F., Pintus G., Al-Asmakh M.A., Abdullah A.M., Nasrallah G.K. (2019). Ecotoxicological assessment of thermally-and hydrogen-reduced graphene oxide/TiO_2_ photocatalytic nanocomposites using the zebrafish embryo model. Nanomaterials.

[65] Posadino A.M., Giordo R., Cossu A., Nasrallah G.K., Shaito A., Abou-Saleh H., Eid A.H., Pintus G. (2019). Flavin Oxidase-Induced ROS Generation Modulates PKC Biphasic Effect of Resveratrol on Endothelial Cell Survival. Biomolecules.

[66] Fois A.G., Posadino A.M., Giordo R., Cossu A., Agouni A., Rizk N.M., Pirina P., Carru C., Zinellu A., Pintus G. (2018). Antioxidant activity mediates pirfenidone antifibrotic effects in human pulmonary vascular smooth muscle cells exposed to sera of idiopathic pulmonary fibrosis patients. Oxid. Med. Cell. Longev..

[67] Pasciu V., Posadino A.M., Cossu A., Sanna B., Tadolini B., Gaspa L., Marchisio A., Dessole S., Capobianco G., Pintus G. (2010). Akt downregulation by flavin oxidase-induced ROS generation mediates dose-dependent endothelial cell damage elicited by natural antioxidants. Toxicol. Sci..

[68] Arbab I.A., Abdul A.B., Sukari M.A., Abdullah R., Syam S., Kamalidehghan B., Ibrahim M.Y., Taha M.M., Abdelwahab S.I., Ali H.M. (2013). Dentatin isolated from Clausena excavata induces apoptosis in MCF-7 cells through the intrinsic pathway with involvement of NF-kappaB signalling and G0/G1 cell cycle arrest: A bioassay-guided approach. J. Ethnopharmacol..

[69] Lackmann C., Santos M.M., Rainieri S., Barranco A., Hollert H., Spirhanzlova P., Velki M., Seiler T.B. (2018). Novel procedures for whole organism detection and quantification of fluorescence as a measurement for oxidative stress in zebrafish (Danio rerio) larvae. Chemosphere.

[70] Kasibhatla S., Amarante-Mendes G.P., Finucane D., Brunner T., Bossy-Wetzel E., Green D.R. (2006). Acridine Orange/Ethidium Bromide (AO/EB) staining to detect apoptosis. CSH Protoc..

